# What Is the Difference? Rereading Shakespeare’s Sonnets —An Eye Tracking Study

**DOI:** 10.3389/fpsyg.2020.00421

**Published:** 2020-03-26

**Authors:** Shuwei Xue, Arthur M. Jacobs, Jana Lüdtke

**Affiliations:** ^1^Department of Experimental and Neurocognitive Psychology, Freie Universität Berlin, Berlin, Germany; ^2^Center for Cognitive Neuroscience Berlin, Berlin, Germany

**Keywords:** rereading, poetry reading, eye movements, QNA, predictive modeling

## Abstract

Texts are often reread in everyday life, but most studies of rereading have been based on expository texts, not on literary ones such as poems, though literary texts may be reread more often than others. To correct this bias, the present study is based on two of Shakespeare’s sonnets. Eye movements were recorded, as participants read a sonnet then read it again after a few minutes. After each reading, comprehension and appreciation were measured with the help of a questionnaire. In general, compared to the first reading, rereading improved the fluency of reading (shorter total reading times, shorter regression times, and lower fixation probability) and the depth of comprehension. Contrary to the other rereading studies using literary texts, no increase in appreciation was apparent. Moreover, results from a predictive modeling analysis showed that readers’ eye movements were determined by the same critical psycholinguistic features throughout the two sessions. Apparently, even in the case of poetry, the eye movement control in reading is determined mainly by surface features of the text, unaffected by repetition.

## Introduction

When to the sessions of sweet silent thought
*I summon up remembrance of things past*,

William Shakespeare, Sonnets 30 (ll. 1-2)

What happens if you read a text for the second time? You may read it faster, remember more details and understand it better. This improvement, widely known as the rereading benefit or rereading effect, has been noted in many studies (see Raney, 2003, for a review). Most of them, however, have been based on the rereading of expository texts (e.g., Hyönä and Niemi, 1990; Levy et al., 1991, 1992; Raney and Rayner, 1995; Raney et al., 2000; Rawson et al., 2000; Schnitzer and Kowler, 2006; Kaakinen and Hyönä, 2007; Margolin and Snyder, 2018), only a few of them on the rereading of literary texts (e.g., Dixon et al., 1993; Millis, 1995; Kuijpers and Hakemulder, 2018) and only one of these on the rereading of poetry (Hakemulder, 2004). None of those based on literary texts used direct or indirect methods to record the cognitive processes associated with comprehension and appreciation while they were happening. Researchers have relied on assessments made by readers after, not during, the process of reading. We wished to overcome this limitation by relying not only on assessments made later but also on eye-movements made during the reading of poetry. Here we shall begin by discussing earlier studies that show the benefit of rereading, go on to present our own approach, put forward hypotheses and finally check them empirically.

### The Effect of Rereading Expository and Literary Texts

Ever since the rereading paradigm was introduced by Hyönä and Niemi (1990), it has been used in a few studies in various domains (e.g., by Levy et al., 1991; Raney et al., 2000; Schnitzer and Kowler, 2006; Kaakinen and Hyönä, 2007). Readers have to read a text more than once, and their way of reading is assessed during or after each session (e.g., by eye tracking or self-assessment). In other studies particular attention was paid to the effect of reading words or phrases repeated within a text (e.g., Kamienkowski et al., 2018), but this is not our concern.

As mentioned above, most studies of rereading have used expository texts as a basis. Expository texts are treated as sources of information stipulating reading processes directed to information intake, so studies using such texts have tended to focus on whether a reader remembers and understands more after the second compared to the first session. The main findings are: firstly, readers who read an expository text twice recalled significantly more than those who read it only once (Amlund et al., 1986; Durgunoǧlu et al., 1993); secondly, rereading facilitated readers to build a better comprehension of the topic (Raney et al., 2000; Rawson et al., 2000; Brown, 2002; Schnitzer and Kowler, 2006; Kaakinen and Hyönä, 2007; Margolin and Snyder, 2018). Meanwhile, researchers were also interested in the influence of rereading on reading fluency, e.g., whether the reading time spent on the text or on single words within that text would be reduced. The answers to these questions were positive. That is, after a first reading, not only was the overall time spent on reading the expository text lowered (Millis and King, 2001), but rereading also improved most eye tracking parameters on the word level: total reading time (the sum of all fixation durations on a certain word) was less, regression time (the sum of fixations on a certain word after the first passage) was less, and the rate of skipping was higher (Hyönä and Niemi, 1990; Raney and Rayner, 1995; Raney et al., 2000; Kaakinen and Hyönä, 2007).

Many studies have confirmed the benefit of rereading, but only a few of them have sought the cause. In general, the rereading benefit may have been due to a change in the roles played by lexical, interlexical or supralexical features in the course of reading. Levy et al. (1992, 1993) have assumed that the rereading benefit could be observed not only when rereading the same text but also when reading another text with a similar meaning or context. They checked this assumption by replacing some words with synonyms, by changing the syntactic structure of the text and by using a paraphrased text in the rereading session. The results confirmed their hypotheses. However, Raney et al. (2000) found that when a paraphrased version of the original text (words from the related texts were replaced by synonyms) was used for the second reading, only gaze duration (the sum of all fixation durations on a certain word during first passage) and total reading time were less. They assumed that rereading had a stronger influence on later processing stages compared to early ones. To clarify at least the role of some lexical features, Raney and Rayner (1995) have tried changing the frequency of words in expository texts, but the decrease in fixation durations was the same for low- and high-frequency words across readings. Likewise, Chamberland et al. (2013) found that the benefit of rereading was the same for content and function words and for low- and high-frequency words, except in the case of gaze duration, when the rereading effect was greater for function than for content words. However, some studies have found that low-frequency words benefit more from multiple readings than high-frequency words (see Kinoshita, 2006, for a review). In other words, results have been inconsistent regarding the effects of rereading on eye tracking parameters in the early stages of the process (e.g., on gaze duration), especially in the case of various psycholinguistic features. The exact roles played by psycholinguistic features on various eye tracking parameters in rereading need further investigation.

Moreover, all the above findings are based on the rereading of expository texts. There have been only a handful studies on rereading of literary texts, and these have relied only on assessments made after reading. Not surprisingly, these studies also found the classical rereading effects, e.g., enhanced comprehension (e.g., Klin et al., 2007; Kuijpers and Hakemulder, 2018). Especially in the case of literary texts, researchers have also been interested in whether rereading affects a reader’s appreciation and aesthetic emotional reactions as a result of ‘literary/foregrounding effects. They assumed that ‘literary/foregrounding effects’ might be related to the level of comprehension (Kuijpers and Hakemulder, 2018), so increased during second reading (Dixon et al., 1993). In line with this hypotheses, the scant studies using literary texts found that rereading indeed influenced readers’ appreciation, insofar as readers tended to rate texts as more likeable after the rereading session (e.g., Dixon et al., 1993; Millis, 1995; Kuijpers and Hakemulder, 2018). The only study on the rereading of poetry has confirmed this hypothesis (Hakemulder, 2004). Nevertheless, none of the studies based on literary texts have checked cognitive and emotional processes associated with comprehension and appreciation while they were happening, by for instance recording the movements of a reader’s eyes on single word level. Whether a literary text is read more fluently the second time round is still an open question.

Hence the main aim of the present study is to examine the effects of rereading poetic texts by using not only assessments made by readers *after* the sessions but also records of eye-movements made *during* the sessions, to find out whether rereading affects a reader’s understanding and appreciation and increases the fluency of reading. A further aim is to check whether surface psycholinguistic features, like *word frequency*, may play a role in changing eye tracking parameters across reading sessions.

### Eye Movement Research on Poetry Reading

As we all know, it is not easy to conduct research using natural texts, as they are mostly very complex (Jacobs et al., 2017; Xue et al., 2017, 2019). Especially if we use literary texts such as poems not specially designed for research (Bailey and Zacks, 2011; Willems and Jacobs, 2016), simple or complex text features seldom occur without interacting with many other features on various levels. Although there have been studies on the reading of literary texts or poems (e.g., Sun et al., 1985; Lauwereyns and d’Ydewalle, 1996; Carrol and Conklin, 2014; Dixon and Bortolussi, 2015; Jacobs et al., 2016a, b; van den Hoven et al., 2016; Müller et al., 2017), the vast majority of eye tracking studies on reading were constrained to experimental textoids and tested only a few selected features while ignoring many others (Rayner et al., 2001; Reichle et al., 2003; Engbert et al., 2005; Rayner and Pollatsek, 2006; Rayner, 2009).

Within the framework of neurocognitive poetics (Jacobs, 2011, 2015a,b; Willems and Jacobs, 2016; Nicklas and Jacobs, 2017), two steps have been suggested to cope with the innumerable features of texts and/or readers and their many (non-linear) interactions. Firstly, a way should be found to break the complex literary works up into simpler, measurable features, for instance by Quantitative Narrative Analysis (QNA; e.g., Jacobs, 2015a, 2017, 2018a, 2019; Jacobs et al., 2016a, 2017; Jacobs and Kinder, 2017, 2018; Xue et al., 2019). Secondly, proper statistical and machine learning modeling tools should be chosen to cope with intercorrelated, non-linear relationships between the many features which may affect the (re)reading of poetry (e.g., Jakobson and Lévi-Strauss, 1962; Schrott and Jacobs, 2011; Jacobs, 2015a,b,c,^2019^; Jacobs et al., 2016a,b).

Recently, a QNA-based predictive approach was successfully applied to account for eye tracking parameters in the reading of three of Shakespeare’s sonnets (sonnet 27, 60, and 66) using multiple psycholinguistic features (Xue et al., 2019). In the study of Xue et al., 2019, seven surface psycholinguistic features, a combination of well-studied (*word length*, *word frequency*, and *higher frequent neighbors*) and less-studied and novel features (*orthographic neighborhood density*, *orthographic dissimilarity*, *consonant vowel quotient*, and *sonority score*), were computed based on the Neurocognitive Poetics Model (NCPM, Jacobs, 2011, 2015a,b; Willems and Jacobs, 2016; Nicklas and Jacobs, 2017) and recent proposals about QNA (e.g., Jacobs, 2017, 2018a,b; Jacobs et al., 2017). In addition, two non-linear interactive approaches, i.e., neural nets and bootstrap forests, were compared with a general linear approach (standard least squares regression), to look for the best way to predict three eye tracking parameters (first fixation duration, total reading time, and fixation probability) using the seven above mentioned features. For the prediction of first fixation duration, none of the three approaches yielded appropriate model fits, as first fixation duration may have been due more to fast and automatic reading behavior rather than to lexical parameters (Hyönä and Hujanen, 1997; Clifton et al., 2007). For the other two parameters, total reading time and fixation probability, neural nets outperformed the general linear approach and also the bootstrap forests. This might be due to the fact, that within this context neural nets could best deal with the complex interactions and non-linearities in the data (Coit et al., 1998; Breiman, 2001; Francis, 2001; Yarkoni and Westfall, 2017). Most importantly, the feature importance analysis of the optimal neural nets approach detected that the two well-known basic features, *word length* and *word frequency*, were most important in accounting for the variance in total reading time and fixation probability. Moreover, also two of the novel features were important predictors. One of the two phonological features, the *sonority score*, was important for predicting both total reading time and fixation probability. *Orthographic neighborhood density* and *orthographic dissimilarity* proved to be important for predicting total reading time, whereas *orthographic neighborhood density* proved to be important for predicting fixation probability.

For the present study, which is a first attempt to evaluate the effects of surface psycholinguistic features in a poetry rereading investigation using eye tracking, we also want to compare the predictive performance of neural nets as an example of a non-linear interactive approach with a general linear approach, including the same seven predictors used in Xue et al. (2019), but with a new larger sample of readers. Thus, in the context of the ‘replication crisis’ debate (Earp and Trafimow, 2015; Maxwell et al., 2015; Shrout and Rodgers, 2018), the present study also served as a replication (Xue et al., 2019), i.e., whether a neural nets approach could build satisfactory models in a rereading study and whether the same ‘important features’ in predicting relevant eye tracking parameters would be detected again.

To summarize, the current study examined the general validity of findings about rereading by using two of Shakespeare’s sonnets. We asked: (1) whether rereading improves understanding and appreciation; (2) whether rereading increases reading fluency; (3) whether the roles of surface features change across reading sessions. We used the terms *first session* and *last session* to denote the two reading sessions, each of which consisted of reading a sonnet then filling in a questionnaire. The terms have been redefined because poems, unlike expository prose, are seldom read straight through from beginning to end (Müller et al., 2017; Xue et al., 2017), so a lot of rereading took place within each session. For the sake of improvement in appreciation (Kuijpers and Hakemulder, 2018), we also updated the rereading paradigm by inserting a paraphrasing session between the two sessions.

### Hypotheses

Previous studies had shown that rereading improved readers’ comprehension and increased their appreciation of literary texts (Dixon et al., 1993; Millis, 1995; Klin et al., 2007; Kuijpers and Hakemulder, 2018), so we expected to get similar results with poetry. In other words, we expected that readers would identify the topic better (showing more understanding) and appreciate the poem more after the last session.

To determine the effect of rereading on fluency, we concentrated on changes of eye tracking parameters on the word level. Mostly, in the case of expository texts, fluency increased after a first reading session (e.g., Levy et al., 1991, 1993), so we expected the same to be true in the case of poetry. However, we also thought that rereading may mostly affect eye tracking parameters related to later stages of processing (e.g., Raney and Rayner, 1995), so regression time and total reading time would be less for the last session. We also expected that the skipping rate in the last session would be higher, lessening the fixation probability. We had no clear expectations about parameters related to early processing, such as first fixation duration and gaze duration, since rereading involves an interplay of several psycholinguistic features, whose effects had not fully been clarified by earlier investigations (Raney and Rayner, 1995; Kinoshita, 2006; Chamberland et al., 2013).

Using poetic materials for reading and rereading, this study aimed to not only replicate effects already evidenced by studies using expository texts but also replicate findings from Xue et al. (2019). They had successfully applied QNA-based predictive modeling approaches to the reading of poetic texts, to cope with the intercorrelated, non-linear relationships between the many text features. Since Xue et al. (2019) indicated that neural nets outperformed bootstrap forests, here we only included one non-linear interactive approach (neural nets) and one general linear approach (standard least squares regression). We expected that neural nets would provide the best fits to the data of the cross-validation test sets.

Moreover, we were also interested in the causes of the rereading effect. For instance, which surface psycholinguistic features may affect reading fluency across sessions? Or may different features affect it in different sessions? There had been no studies of most of them, so in this sense our study was exploratory.

## Materials and Methods

### Participants

English native speakers were recruited through an announcement released at the Freie Universität Berlin. Altogether 25 people took part (eleven females; *M*_age_ = 23.9 years, *SD*_age_ = 4.3, age range: 19–33 years). They were neither trained literature scholars of poetry nor aware of the purpose of the experiment. All speakers had normal or corrected-to-normal vision and gave their informed, written consent before taking part. They were given eight euros as compensation. This study followed the guidelines of the ethics committee of the Department of Education and Psychology at the Freie Universität Berlin. Some eye movement data were removed, as the eye tracker had failed to record them in full. The data finally used for analyzing the eye movements and predictive modeling came from 22 participants for sonnet 27 (11 females; *M*_age_ = 23.45 years, *SD*_age_ = 4.1, age range: 19–32 years) and 23 participants for sonnet 66 (nine females; *M*_age_ = 24.22 years, *SD*_age_ = 4.36, age range: 19–33 years).

### Apparatus

Eye movements were collected by a remote EYELINK eye tracker (SR Research Ltd., Mississauga, ON, Canada). The sampling frequency was 1000 Hz, and only the right eye was tracked. Readers heads were kept still by a chin-and-head rest. Stimulus presentation was controlled by Eyelink Experiment Builder software (version 1.10.1630)^1^. Stimuli were presented on a 19-inch LCD monitor with a refreshment rate of 60 Hz and a resolution of 1,024 × 768 pixels, 50 cm away from the reader. Each tracking session began with a standard 9-point calibration and validation procedure, to ensure a spatial resolution error of less than 0.5° of the angle of vision.

### Materials

For this rereading experiment, only two of the three Shakespeare’s sonnets used by Xue et al. (2019) were presented, to let readers concentrate without getting tired. The two sonnets were: 27 (‘Weary with toil…’) and 66 (‘Tired with all these…’). Both sonnets covered different topics, “love as tension between body and soul” (sonnet 27) and “social evils during the period Shakespeare lived” (sonnet 66). To increase statistical power for all levels of analysis we collapsed the data across the two sonnets.

### Procedure

The reading was done in a quiet and dimly lit room and consisted of two tasks: the general mood state task and the main task. Readers were told about the whole procedure at the start.

The general mood state task was used at the beginning and at the end of the experiment, to assess any changes in reader’s moods. They were asked to fill in an English version of the German multidimensional mood questionnaire (MDBF; Steyer et al., 1997), to let three bipolar dimensions of subjective feeling (depressed vs. elevated, calmness vs. restlessness, sleepiness vs. wakefulness) on a 7-point rating scale be assessed. The results showed that they were in a neutral mood of calmness and wakefulness throughout. According to the results of paired-simples *t*-tests, there was no significant change of mood before and after the experiment [all *t*(24)s < 2, *p*s > 0.1], as if reading sonnets caused no lingering changes in the global dimensions assessed by MDBF.

The main task was made up of five parts: (a) a first reading session in front of the eye tracker; (b) a paper–pencil task for the first session; (c) an oral paraphrasing line by line; (d) a last reading session in front of the eye tracker; (e) a paper–pencil task for the last session. For the first session, participants were free to read the sonnet at their own speed. Rereading in the course of one session were allowed. Before each sonnet appeared onscreen, readers were presented with a black dot fixation marker (0.6° of the angle of vision) to the left of the first word in line 1, the distance between the dot and first word being 4.6°. When they fixated on the marker, the sonnets appeared automatically. After the first session, readers went to another desk to fill in our self-developed paper-pencil task (see Papp-Zipernovszky et al., unpublished). They got no feedback to their answers. Following this step, they orally paraphrased the sonnet, line by line, according to their own understanding, and again, no feedback or fixed answer was given by the experimenter. The paraphrasing process was recorded by a digital voice recorder. Readers were then asked to reread the sonnet at their own speed before the eye tracker again. Before the last reading session, recalibration was needed. At the end, readers worked on the paper–pencil task for the second time. After answering the questionnaire for the first sonnet, they went on to read the second sonnet in front of the eye tracker. The two sonnets were presented left-aligned in the center of the monitor (distance: 8.0° from the left margin of the screen) by using a font (Arial) with a variable width and a letter size of 22-points (approximately 4.5 × 6.5 mm, 0.5° × 0.7° of the angle of vision). One reader would be shown sonnet 27 first and the next be shown sonnet 66 first and so on, to cancel out any effect due to the sequence. Likewise, a questionnaire was presented before the last session, so a sample questionnaire was also presented before the first.

**FIGURE 1:**
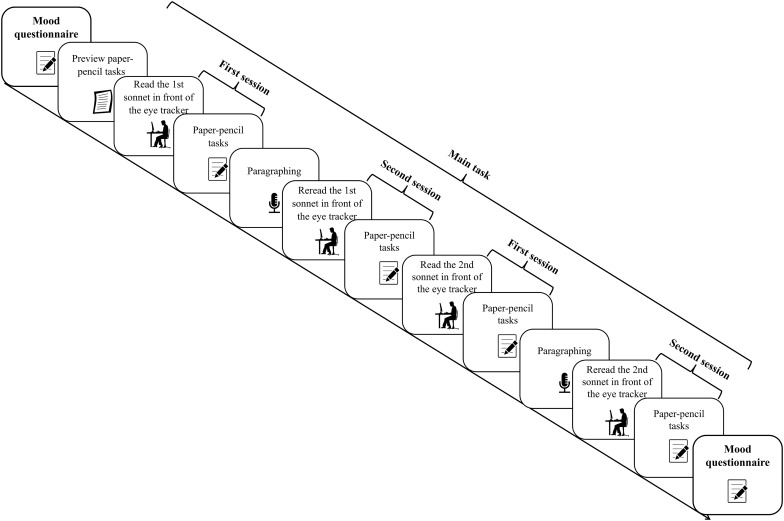
The procedure of the experiment. “1st” and “2nd” refer to the first and second sonnet.

Altogether, the experiment took about 50 min (see Figure 1 for an illustration of the procedure).

### Data Analysis

#### Paper–Pencil Task

Unlike in the paper-pencil task used by Xue et al. (2019) and Papp-Zipernovszky et al. (unpublished), the question about rhyme pairs was included in only the questionnaire used for the last session, so as not to divert attention from comprehension, so in this respect there could be no comparison between the first and the last session. Otherwise, all parts of the questionnaire were the same for the first and last session.

In the present study, we focused on three questions, one related to the general willingness to do any rereading, another one related to comprehension and a third one related to appreciation. Since a lot of “rereading” was involved in reading the questionnaire presented after each session, the question about willingness (“I would like to read this poem again”) was used as a control question. After the last session, participants should have reported less willingness to do any rereading, in being weary and less motivated. The question, “I like this poem,” was used to evaluate participants’ appreciation of it (Lüdtke et al., 2014; Kraxenberger and Menninghaus, 2017). For both questions, readers indicated their agreement with the statements on a 5-point rating scale ranging from 1 = totally disagree to 5 = totally agree. The topic identification question was meant to find out whether readers successfully grasped the main topic of each poem (“Which is the main topic of this poem?”). Six choices were offered, but only one was right. If readers agreed with none of the choices, they could put forward another, which was later evaluated by two experts from the humanities. In the two sessions, 20% of the answers were formulated by the participants themselves (first session: 10 answers; last session: 10 answers). Answers which were not clear or did not exceed the explanation of surface meaning, were evaluated as wrong. For instance, for sonnet 27, answers like “Never resting” or “A journey both physically and mentally” were coded as wrong. None of the self-formulated answers in the first session were right, but 40% of them (4 answers) were right in the last session.

JMP 14 Pro^2^ was used for the statistical analyses. For the two questions about appreciation and a general willingness to do any rereading, we used paired-samples *t*-tests, to check the differences between the first session and the last. Since we evaluated and recoded the answers for the topic identification question as “yes” or “no” (categorical variable), we then used a non-parametric test, i.e., Bowker’s test, to check the difference between sessions.

#### Eye Tracking Parameters

Pre-processing of the raw data was done by EyeLink Data Viewer^3^. As mentioned earlier, data from three readers of sonnet 27 and from two readers of sonnet 66 were removed, because the eye tracker had failed to record their eye movements. From the data, we then determined first fixation duration (the duration of first fixation on a certain word), gaze duration (the sum of all fixations on a certain word during first passage), regression time (the sum of fixations on a certain word after first passage) and total reading time (the sum of all fixation durations on a certain word) for each word, participant and sonnet.

For all analyses predicting eye tracking parameters, we focused on the effect of text-based features on rereading. We also decided to use the same pre-processed data for all analyses. To reliably test the effect of the surface features used in Xue et al. (2019) in predicting eye tracking parameters in first and last reading by neural nets, the eye tracking data have to be aggregated at the word level. We therefore cumulated the data over all participants to obtain the mean values for each word within each sonnet and each session. In order to take the amount of skipping into account, fixation probability was calculated. Skipped words were thus treated as missing values (skipping rate: *M*_first–session_ = 13%, *SD*_first–session_ = 0.34; *M*_last–session_ = 20%, *SD*_last–session_ = 0.40). For instance, in the last session, words fixated by all participants, like ‘expired’ (sonnet 27) or ‘jollity’ (sonnet 66) had a probability of 100%, whereas words fixated by only one or two participants like ‘To’ (sonnet 27) or ‘I’ (sonnet 66) had fixation probabilities below 20%. Altogether, in the first session over 38% of the words had a fixation probability of 100% and in the last session the amount decreased to 25%, which led to a highly asymmetric distribution. However, unlike Xue et al. (2019), we did not aggregate the eye tracking data for words appearing twice or more often. Instead, here we included positional information (line number: *lineNo.*; word number in each line: *wordNo.*) as a feature in the predictive modeling analysis. For each reading session the total sample size entering in the models was *N* = 202 words. The correlations between the five aggregated eye tracking parameters are shown in Table 1.

**TABLE 1:** Correlations between the five eye tracking parameters.

Session	Variables	1	2	3	4	5
First	(1) First fixation duration	–				
	(2) Gaze duration	0.65	–			
	(3) Regression time	0.14	0.46	–		
	(4) Total reading time	0.34	0.72	0.95	–	
	(5) Fixation probability	0.16	0.34	0.62	0.61	–
Last	(1) First fixation duration	–				
	(2) Gaze duration	0.77	–			
	(3) Regression time	0.20	0.45	–		
	(4) Total reading time	0.53	0.82	0.88	–	
	(5) Fixation probability	0.23	0.44	0.55	0.59	–

To test for the rereading effects at the word-level, linear mixed models (LMM) with one fixed effect (session) and one random effect (word nested within sonnet) were applied to the five eye tracking parameters using JMP 14 Pro.

#### Predictors for Predictive Modeling

##### Positional Information

As mentioned earlier, several words are repeated in the sonnets (e.g., mind), so we added the positional information (*lineNo.* and *wordNo.*) of the words in each sonnet.

##### Psycholinguistic Features

Seven psycholinguistic features were calculated for all words (word-token, 202 words) in the two sonnets: *word length* (*wl*) is the number of letters per word; *word frequency* (*logf*) is the log transformed number of times that a word appears in the Gutenberg Literary English Corpus as a reference (GLEC; Jacobs, 2018b; Xue et al., 2019); *orthographic neighborhood density* (*on*) is the number of words of the same length as a certain word and differing by only one letter in GLEC; *higher frequent neighbors* (*hfn*) is the number of orthographic neighbors with a higher word frequency than the word in GLEC; *orthographic dissimilarity* (*odc*) is the word’s mean Levenshtein distance from all other words in the corpus (GLEC), a metric that generalizes orthographic similarity to words of different lengths; *consonant vowel quotient* (*cvq*) is the quotient of consonants and vowels in one word; *sonority score* (*sonscore*) is the sum of phonemes’ sonority hierarchy with a division by the square root of *wl* (the sonority hierarchy of English phonemes yields 10 ranks: [a] > [e o] > [i u j w] > [r] > [l] > [m n η] > [z v] > [f θ s] > [b d g] > [p t k]; Clements, 1990; Jacobs and Kinder, 2018). For example, in our two sonnets, ART got the *sonscore* of 10 × 1 [a] + 7 × 1 [r] + 1 × 1 [t] = 18/SQRT (3) = 10.39. As shown in Table 2, some of these psycholinguistic features were highly correlated, hence the need to apply machine-learning tools in a predictive approach (e.g., Coit et al., 1998; Francis, 2001; Tagliamonte and Baayen, 2012; Yarkoni and Westfall, 2017).

**TABLE 2:** Correlations between the seven psycholinguistic features.

Variables	1	2	3	4	5	6	7
(1) Word length (*wl*)	–						
(2) Log frequency (*logf*)	−0.81	–					
(3) Orthographic neighbors (*on*)	−0.85	0.72	–				
(4) Higher frequency neighbors (*hfn*)	−0.23	−0.06	0.28	–			
(5) Orthographic dissimilarity based on corpus (*odc*)	0.62	−0.44	−0.28	−0.12	–		
(6) Consonant vowel quotient (*cvq*)	0.30	−0.10	−0.36	−0.14	0.04	–	
(7) Sonority score (*sonscore*)	0.74	−0.64	−0.61	−0.23	0.58	0.07	–

#### Predictive Modeling

We also utilized the JMP 14 Pro to run all predictive modeling analyses^4^. As described above, nine predictors (*lineNo.*, *wordNo.*, *wl*, *logf*, *on*, *hfn*, *odc*, *cvq*, and *sonscore*) and five eye tracking parameters (first fixation duration, gaze duration, regression time, total reading time, and fixation probability) were included in these analyses. The values of all eye movement parameters and psycholinguistic features were standardized before being analyzed in predictive modeling.

Cross-validation was used as a solution to the problem of overfitting. Among the methods of cross-validation, K-fold appears to work better than hold-out in the case of small sample size, because it uses data more efficiently (Refaeilzadeh et al., 2009). It divides the original data into K subsets. In turn, each of the K sets is used to test the model fit on the rest of the data, fitting a total of K models. The model giving the best test statistic is chosen as the final model. The 10-fold cross-validation is usually recommended as the best method, since it provides the least biased estimation of the accuracy (Kohavi, 1995). Therefore, in the present study, instead of the 10% hold-out cross-validation method (i.e., taking 90% of the data as a training set and the remaining 10% as a test set) used in Xue et al. (2019), we used 10-fold cross-validation.

Given the intrinsic probabilistic nature of neural nets, predictive modeling results vary across repeated runs. These differences depend also on the splitting into training and test set during cross-validation (total sample size = 202 words, i.e., about 20 cases in each fold during cross-validation). To cover potential disadvantages of splitting small samples, the k-fold cross-validation procedure was repeated 100 times and the model fit scores were averaged (e.g., Were et al., 2015). Note that for the standard least squares regression, JMP 14 Pro only provides the 100 model fit scores for the test sets, which, of course, is the relevant piece of information.

Following the procedure of Xue et al. (2019) for comparing neural nets and linear regression, our criterion for a satisfactory model fit score was a mean *R*^2^ > 0.30 and a low *SD*). When the non-linear interactive approach proved to be satisfactory, we determined feature importance (*FI*), an index of effect strength used in machine learning^5^. In the current study, *FI*s were computed as the total effect of each predictor as assessed by the *dependent resampled inputs* option of the JMP14 Pro software. The total effect is an index quantified by sensitivity analysis, reflecting the relative contribution of a feature both alone and together with other features (for details, see also Saltelli, 2002). This measure is interpreted as an ordinal value on a scale of 0 to 1, *FI* values > 0.1 being considered as ‘important’ (cf. Strobl et al., 2009). If the general linear approach proved to be satisfactory, the parameter estimates were reported instead of *FI*s.

## Results

### Paper–Pencil Task

**FIGURE 2:**
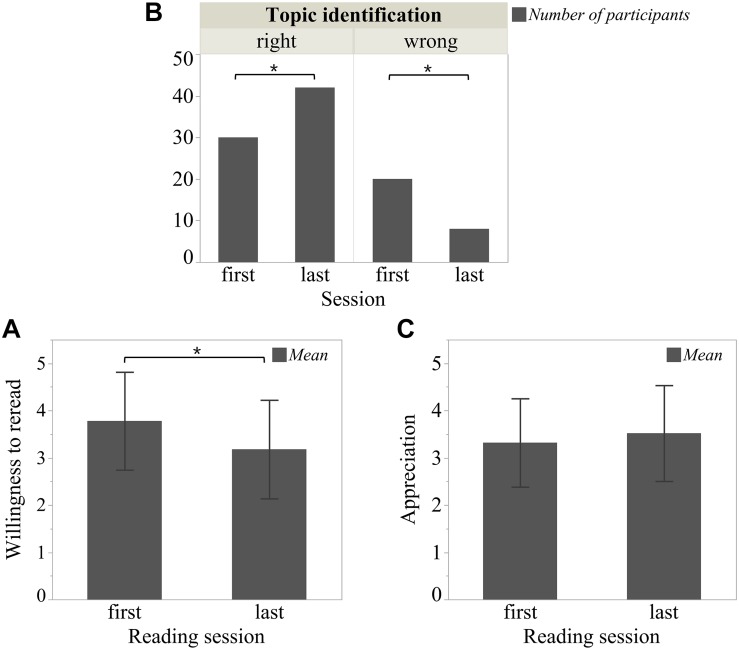
Rereading effect on rating data. **(A)** “Willingness to do any rereading,” **(B)** “Topic identification,” and **(C)** “Appreciation” were separately collected from three questions: “I would like to read this poem again,” “Which is the main topic of this poem,” and “I like this poem.” For questions related to “Willingness to do any rereading” and “Appreciation,” readers indicated their agreement with the statements on a 5-point rating scale ranging from 1 = totally disagree to 5 = totally agree. For the topic identification question, six choices were offered, but only one was right. If readers agreed with none of the choices, they could put forward another, which was later evaluated by two experts from the humanities. *p < 0.05. Error bar is constructed using one standard deviation from the mean.

The results of the rereading effects on rating data are shown in Figure 2: Firstly, there was a significant effect on readers’ willingness to do any rereading [*t*(49) = 3.32, *p* = 0.002]. After the last session, readers were less willing to reread the sonnet than after the first session (first session: *M* = 3.78, *SD* = 1.04; last session: *M* = 3.18, *SD* = 1.04). Secondly, the rereading effect on topic identification was also significant (χ^2^ = 8, *df* = 1, *p* = 0.005). Readers were more able to choose the right topic after the last session than after first session (first session: *N*_right_ = 30, *N*_wrong_ = 20; last session: *N*_right_ = 42, *N*_wrong_ = 8).

Readers tended to appreciate a sonnet in the last session more than in the first (first session: *M* = 3.32, *SD* = 0.94; last session: *M* = 3.52, *SD* = 1.02), but the difference was not statistically significant [*t*(49) = −1.81, *p* = 0.077]. We also checked for each sonnet separately by applying a paired-samples *t=*test. For sonnet 27, there was no significant difference in appreciation, whether it was read in the first or last session [*t*(24) = −0.30, *p* = 0.77; first session: *M* = 3.88, *SD* = 0.67; last session: *M* = 3.92, *SD* = 0.81], but there was a significant difference for sonnet 66 [*t*(49) = −2.09, *p* = 0.047], which was appreciated more if read in the last session (first session: *M* = 2.76, *SD* = 0.83; last session: *M* = 3.12, *SD* = 1.05).

### Eye Tracking Parameters

**FIGURE 3:**
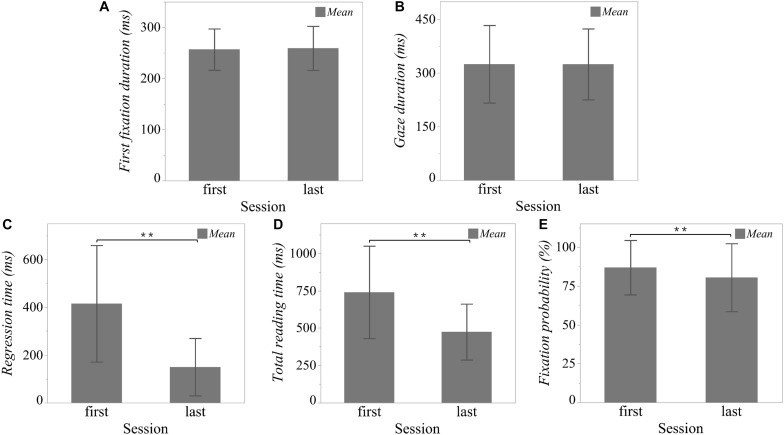
Rereading effect on eye tracking parameters. To test for the rereading effects on word-level eye tracking parameters, linear mixed models (LMM) with one fixed effect (session) and one random effect (word nested within sonnet) were applied to the five eye tracking parameters **(A)** “First fixation duration,” **(B)** “Gaze duration,” **(C)** “Regression time,” **(D)** “Total reading time,” **(E)** “Fixation probability”. ** *p* < 0.01. Error bar is constructed using one standard deviation from the mean.

As illustrated in Figure 3, linear mixed models (LMM) with one fixed effect (session) and one random effect (word nested within sonnet) showed significant rereading effects on regression time [*t*(1) = 22.34; *p* < 0.0001], total reading time [*t*(1) = 20.28; *p* < 0.0001], and fixation probability [*t*(1) = 6.54; *p* < 0.0001]. In the last session as compared to the first, readers tended to spend less time on regressions (first session: *M* = 414.90 ms, *SD* = 243.78; last session: *M* = 149.85 ms, *SD* = 120.12) and to shorten their total reading time (first session: *M* = 739.80 ms, *SD* = 309.48; last session: *M* = 474.45 ms, *SD* = 187.05). Moreover, the probability of fixating on a word was likewise smaller in the last session (first session: *M* = 86.81%, *SD* = 17.49; last session: *M* = 80.35%, *SD* = 21.85).

However, for first fixation duration (first session: *M* = 256.84 ms, *SD* = 40.43; last session: *M* = 259.25 ms, *SD* = 43.04) and gaze duration (first session: *M* = 324.91 ms, *SD* = 108.51; last session: *M* = 324.60 ms, *SD* = 99.19), we found no significant differences between the two sessions [first fixation duration: *t*(1) = −0.83; *p* = 0.41; gaze duration: *t*(1) = 0.06; *p* = 0.95].

### Predictive Modeling

**FIGURE 4:**
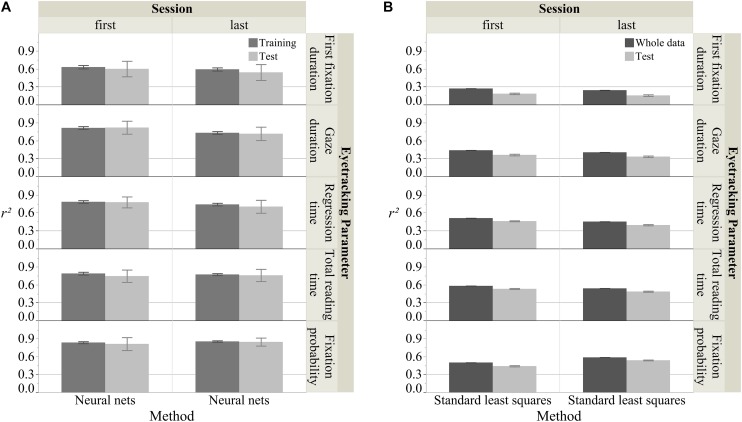
Fit scores for different models and measures. For neural nets **(A)**, *R*^2^s from 100 iterations were averaged for both the training and test sets. For standard least squares regressions **(B)**, the *R*^2^ for the whole data set and the mean *R*^2^s from 100 iterations for the test sets were calculated. Nine predictors (*lineNo.*, *wordNo.*, *wl*, *logf*, *on*, *hfn*, *odc*, *cvq*, and *sonscore*) and five response parameters (first fixation duration, gaze duration, regression time, total reading time, and fixation probability) were included in analyses. Each error bar is constructed using one standard deviation from the mean.

**FIGURE 5:**
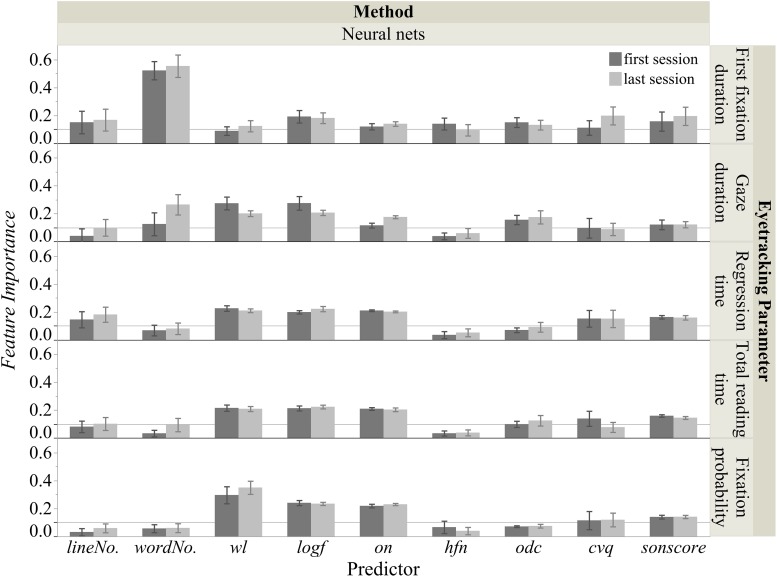
Feature importance for the five eye tracking parameters.

Figure 4 shows the overall *R*^2^ (100 iterations) for predicting the five eye tracking parameters using the two modeling approaches. As mentioned above, for the standard least squares regression the *R*^2^ for the whole data set and the mean *R*^2^ for the test sets was computed. As illustrated in Figure 4, generally neural nets produced acceptable models for all five eye tracking parameters (mean *R*^2^ > 0.30), and they also produced much higher model fits than standard least squares regression. Therefore, the nine *FI*s for the neural nets were computed (see Figure 5). Below we illustrate our results for the five eye tracking parameters, respectively.

#### First Fixation Duration

As shown in Figure 4, in the first session, neural nets produced good fits for both the training and test sets (mean *R*^2^_*train*_ = 0.63, *SD R*^2^_*train*_ = 0.03; mean *R*^2^_*test*_ = 0.60, *SD R*^2^_*test*_ = 0.13) in contrast to standard least squares (*R*^2^_*whole*_ = 0.27; mean *R*^2^_*test*_ = 0.18, *SD R*^2^_*test*_ = 0.01). The same was true for the last session. Only neural nets produced good fits (neural nets: mean *R*^2^_*train*_ = 0.59, *SD R*^2^_*train*_ = 0.03; mean *R*^2^_*test*_ = 0.54, *SD R*^2^_*test*_ = 0.13; standard least squares: mean *R*^2^_*whole*_ = 0.24; mean *R*^2^_*test*_ = 0.16, *SD R*^2^_*test*_ = 0.01).

The *FI* analysis of the optimal neural nets approach in Figure 5 suggested that in the first session nearly all the predictors were important for predicting first fixation duration (*wordNo.* [0.52], *logf* [0.19], *sonscore* [0.16], *lineNo.* [0.15], *odc* [0.15], *hfn* [0.14], *on* [0.12], *cvq* [0.11]), except for *wl* (0.09). Similarly, in the last session also all predictors were important (*wordNo.* [0.55], *cvq* [0.20], *sonscore* [0.20], *logf* [0.18], *lineNo.* [0.17], *on* [0.14], *odc* [0.13], *wl* [0.12], *hfn* [0.10]).

#### Gaze Duration

Figure 4 shows that in the first session, neural nets and standard least squares both produced acceptable fits, but those of neural nets were clearly higher (mean *R*^2^_*train*_ = 0.82, *SD R*^2^_*train*_ = 0.02; mean *R*^2^_*test*_ = 0.82, *SD R*^2^_*test*_ = 0.11) than standard least squares (*R*^2^_*whole*_ = 0.44; mean *R*^2^_*test*_ = 0.36, *SD R*^2^_*test*_ = 0.01). The same was true for the last session. Although both approaches yielded acceptable models, neural nets again produced clearly better fits (mean *R*^2^_*train*_ = 0.73, *SD R*^2^_*train*_ = 0.02; mean *R*^2^_*test*_ = 0.72, *SD R*^2^_*test*_ = 0.11) than standard least squares (*R*^2^_*whole*_ = 0.41; mean *R*^2^_*test*_ = 0.33, *SD R*^2^_*test*_ = 0.01).

The *FI* analysis of the optimal neural nets approach shown in Figure 5 suggested that in the first session, seven predictors were important for predicting gaze duration (*logf* [0.27], *wl* [0.27], *odc* [0.16], *wordNo.* [0.12], *sonscore* [0.12], *on* [0.11], *cvq* [0.10]), while *lineNo.* (0.04) and *hfn* (0.04) were less important. For the last session, there were also seven important predictors (*wordNo.* [0.26], *logf* [0.21], *wl* [0.20], *on* [0.18], *odc* [0.17], *sonscore* [0.12], *lineNo.* [0.10]), while this time the less important ones were *cvq* (0.09) and *hfn* (0.06).

#### Regression Time

As illustrated in Figure 4, similar to gaze duration, in the first session, neural nets and standard least squares again were both acceptable; but neural nets produced higher model fits (mean *R*^2^_*train*_ = 0.78, *SD R*^2^_*train*_ = 0.02; mean *R*^2^_*test*_ = 0.78, *SD R*^2^_*test*_ = 0.09) than standard least squares (*R*^2^_*whole*_ = 0.51; mean *R*^2^_*test*_ = 0.46, *SD R*^2^_*test*_ = 0.01). The same was true for the last session with neural nets (mean *R*^2^_*train*_ = 0.74, *SD R*^2^_*train*_ = 0.02; mean *R*^2^_*test*_ = 0.70, *SD R*^2^_*test*_ = 0.11) being better than standard least squares (*R*^2^_*whole*_ = 0.45; mean *R*^2^_*test*_ = 0.40, *SD R*^2^_*test*_ = 0.01).

Figure 5 shows the *FI* analysis of the optimal neural nets approach suggesting that in the first session, six predictors were important for regression time (*wl* [0.23], *on* [0.21], *logf* [0.20], *sonscore* [0.16], *cvq* [0.15], *lineNo.* [0.15]), while *odc* (0.07), *wordNo.* (0.07), and *hfn* (0.04) were less important. For the last session, the important predictors were the same (*logf* [0.22], *wl* [0.21], *on* [0.20], *lineNo.* [0.18], *sonscore* [0.16], *cvq* [0.15]), as were the less important ones: *odc* (0.09), *wordNo.* (0.08), and *hfn* (0.05).

#### Total Reading Time

Likewise, Figure 4 shows results for neural nets (mean *R*^2^_*train*_ = 0.79, *SD R*^2^_*train*_ = 0.02; mean *R*^2^_*test*_ = 0.75, *SD R*^2^_*test*_ = 0.10) and standard least squares (*R*^2^_*whole*_ = 0.58; mean *R*^2^_*test*_ = 0.53, *SD R*^2^_*test*_ = 0.01) during the first session and for the last session: neural nets (mean *R*^2^_*train*_ = 0.77, *SD R*^2^_*train*_ = 0.02; mean *R*^2^_*test*_ = 0.76, *SD R*^2^_*test*_ = 0.10) and standard least squares (*R*^2^_*whole*_ = 0.54; mean *R*^2^_*test*_ = 0.49, *SD R*^2^_*test*_ = 0.01).

The *FI* analysis of the optimal neural nets approach shown in Figure 5 suggested that in the first session, six predictors were important for total reading time (*wl* [0.22], *logf* [0.21], *on* [0.21], *sonscore* [0.16], *cvq* [0.14], *odc* [0.10]), while *lineNo.* (0.08), *hfn* (0.03), and *wordNo.* (0.03) were less important. For the last session, there were also six important predictors (*logf* [0.22], *wl* [0.21], *on* [0.20], *sonscore* [0.14], *odc* [0.12], *lineNo.* [0.10]), and three less important ones: *wordNo.* (0.09), *cvq* (0.08), and *hfn* (0.04).

#### Fixation Probability

Finally, Figure 4 also gives results for the first session for both neural nets (mean *R*^2^_*train*_ = 0.84, *SD R*^2^_*train*_ = 0.02; mean *R*^2^_*test*_ = 0.81, *SD R*^2^_*test*_ = 0.02) and standard least squares (*R*^2^_*whole*_ = 0.50; mean *R*^2^_*test*_ = 0.44, *SD R*^2^_*test*_ = 0.01). For the last session, again, neural nets produced better model fits (mean *R*^2^_*train*_ = 0.86, *SD R*^2^_*train*_ = 0.01; mean *R*^2^_*test*_ = 0.85, *SD R*^2^_*test*_ = 0.07) than standard least squares (*R*^2^_*whole*_ = 0.59; mean *R*^2^_*test*_ = 0.54, *SD R*^2^_*test*_ = 0.01).

The *FI* analysis of the optimal neural nets approach in Figure 5 suggested that in the first session, five predictors were important for fixation probability (*wl* [0.30], *logf* [0.24], *on* [0.22], *sonscore* [0.14], *cvq* [0.11]), while *odc* (0.07), *hfn* (0.07), *wordNo.* (0.06), and *lineNo.* (0.03) were less important. For the last session, the important predictors were the same (*wl* [0.35], *logf* [0.23], *on* [0.23], *sonscore* [0.14], *cvq* [0.12]), as were the less important ones: *odc* (0.07), *wordNo.* (0.06), *lineNo.* (0.06), and *hfn* (0.04).

## Discussion

Every day we all read many kinds of texts such as news reports, blogs, brochures, biographies, reviews, instructions and regulations, novels or poetry for the sake of being informed or entertained. Usually, we read a text or parts of a text more than once to grasp all the main points or to deepen our enjoyment, and this is especially true in the case of literature. Once a text is familiar, after a first reading, it may be read faster. All of these effects are familiar and are known as the classical reading benefit found in many studies based on expository texts, but few examined literary texts such as poetry. Arguably no writer of classical literature is more eminent than Shakespeare, so we chose two of his sonnets as our materials. We compared the rating data and the eye tracking data in the first session with those in the latter and analyzed the difference, then we also analyzed the roles played by seven surface psycholinguistic features in predicting five eye tracking measures in both sessions with the help of predictive modeling.

### The Rereading Benefit or Rereading Effect

In line with previous studies (e.g., Hakemulder, 2004; Kuijpers and Hakemulder, 2018), our questionnaire data indicated that readers identified the main topic more reliably after the last session. This shows that rereading Shakespeare’s sonnets does indeed enhance readers’ understanding. As assumed by Hyönä and Niemi (1990), a first reading conjures up in readers a mental representation, which rereading may activate for the sake of easier understanding, even in the case of poetry. Moreover, as shown by answers to the question about their willingness to read the poem again, readers were less willing to do so after the last session. Each sonnet involved a lot of rereading, so readers may have felt more fatigue after the last session.

Unlike former studies (e.g., Dixon et al., 1993; Millis, 1995; Kuijpers and Hakemulder, 2018), in our study rereading did not significantly affect readers’ appreciation. However, when we checked the results for each sonnet separately, the effect reappeared, insofar as readers liked sonnet 66 slightly more after the last session than after the first (first session: *M*_*sonnet*66_ = 2.76, *SD*_*sonnet*66_ = 0.83; last session: *M*_*sonnet*66_ = 3.12, *SD*_*sonnet*66_ = 1.05). For sonnet 27 the difference was not significant, however, (first session: *M*_sonnet27_ = 3.88, *SD*_sonnet27_ = 0.67; last session: *M*_sonnet27_ = 3.92, *SD*_sonnet27_ = 0.81). Whether this difference is the result of a ceiling effect (sonnet 27 was already well appreciated after the first session) or the result of different levels of general comprehensibility (sonnet 66 has longer and less frequent words than sonnet 27, e.g., standardized word length: *M*_*sonnet*66_ = 0.24, *SD*_sonnet66_ = 1.10; *M*_sonnet27_ = −0.20, *SD*_sonnet27_ = 0.87; standardized word frequency: *M*_*sonnet*66_ = −0.18, *SD*_sonnet66_ = 1.13; *M*_sonnet27_ = 0.15, *SD*_sonnet27_ = 0.86) has to be tested in future studies.

Besides assessing reading behavior by ratings, we also applied eye tracking as an indirect online method to measure ongoing cognitive and affective processes associated with comprehension and appreciation. Linear mixed model analyses confirmed that rereading increases reading fluency, even in the case of poetry, as shown by a decrease in regression time and total reading time, which are typical of later stages of the process of reading and comprehension. The skipping rate was likewise higher in the last session, so the probability of fixating on any word was smaller during the last session. Rereading seemed to have no effect on first fixation and gaze durations, though. As already mentioned, analysis of eye tracking parameters associated with early stages of the process have not led to consistent findings, especially when various psycholinguistic features were taken into account (Raney et al., 2000; Kinoshita, 2006; Chamberland et al., 2013). In our study, first fixation and gaze durations were nearly the same in the last session as in the first, likely because these parameters reflect fast and automatic initial word recognition processes (cf. Hyönä and Hujanen, 1997; Clifton et al., 2007) hardly affected by rereading.

### QNA-Based Predictive Modeling Approaches

By using machine-learning tools, complex relationships in and between data sets can be disentangled and identified (e.g., Coit et al., 1998; Breiman, 2001; Francis, 2001; Tagliamonte and Baayen, 2012; LeCun et al., 2015; Yarkoni and Westfall, 2017). Among the many machine-learning tools, neural nets may be the most suitable for psychological studies, since they make use of an architecture inspired by the neurons in the human brain (LeCun et al., 2015). In neural nets, data are transmitted from an input layer over one or more hidden layer(s) to the output layer, assigning different weights to all connections between layers during the learning/training phase. The neural nets’ hidden layer(s) also performs a dimension reduction on correlated predictors. Therefore, the approach appears advantageous for studies on natural reading in which multiple psycholinguistic and context features may play a role (Jacobs, 2015a, 2018a). In Xue et al. (2019), the neural nets approach proved to be the optimal one in predicting two eye tracking parameters (total reading time and fixation probability) using seven surface features.

In the present study we successfully replicated the findings of Xue et al. (2019) about reading Shakespeare’s sonnets: (1) the neural nets approach was the best way to predict total reading time and fixation probability using a set of nine psycholinguistic features; (2) *word length*, *word frequency*, *orthographic neighborhood density* and *sonority score* were most important in predicting total reading time and fixation probability for poetry reading, and *orthographic dissimilarity* proved to be important for total reading time. Nevertheless, comparing the results of this study with those of Xue et al. (2019) uncovers some differences. In this present rereading study the *consonant vowel quotient* was also indicated as a potentially important feature for total reading time (first session) and fixation probability (first and last session). This finding of two important phonological features, *sonority score* and *consonant vowel quotient*, is in line with the assumption that consonant status and sonority also play a role in silent reading (Maïonchi-Pino et al., 2008; Berent, 2013), especially of poetic texts (Kraxenberger, 2017).

In contrast to Xue et al. (2019), neural nets also produced acceptable model fits for first fixation duration. That was also true for gaze duration and regression time, two eye tracking parameters not tested in Xue et al. (2019). For all three parameters, neural nets outperformed the standard least square analysis. The calculation of the *FI*s indicated that *word length*, *word frequency*, *orthographic neighborhood density* and *sonority score* were important in predicting first fixation duration, gaze duration and regression time for poetry reading, except that *word length* was less important for predicting first fixation duration in the first reading session (*FI* = 0.09). Crucially, we found that the positional information, i.e., word number in a certain line, was important in predicting first fixation and gaze durations, which again supports the idea that these measures reflect fast and automatic reading behavior and are less sensitive to lexical features (Hyönä and Hujanen, 1997; Clifton et al., 2007).

By applying the predictive modeling approach, we also wanted to find out which psycholinguistic features may cause potential differences in eye tracking parameters for the first and last sessions. The comparison of five eye tracking parameters for first and last reading indicated a significant decrease in regression time, total reading time and fixation probability for the last session. More interestingly, the basic features which were most important in the first session were also the most important ones in the last. Surface features like *word length*, *word frequency*, *orthographic neighborhood density*, and *sonority* thus seem to be basic to eye movement behavior in reading and remain so, no matter how many times a text is read. However, since most of the surface features important in one session were also important in the other, it remains unclear why total reading and regression times decreased in the last session. Perhaps this was due to changes in the importance of other lexico-semantic or complex interlexical and supralexical features (e.g., syntactic complexity; Lopopolo et al., 2019) across reading sessions. As illustrated in Figure 4, the overall model fits were slightly decreased across sessions for all eye tracking parameters except for fixation probability. This could indicate that while surface features play a lesser role, other features become more important, leaving a lot to explore in future research on eye movements in poetry reading.

In conclusion, by using a rereading paradigm, we examined the effects of reading and rereading Shakespeare’s sonnets. Besides assessing reading behavior by rating and examining cognitive processes by using the eye tracking technique, we also checked the roles of surface psycholinguistic features across reading sessions by using predictive modeling. Our study confirmed not only the benefit of rereading a text usually obtained with non-literary materials, but also the advantages of neural nets modeling, as well as the key importance of surface psycholinguistic features in all sessions of reading.

## Limitations and Outlook

In this study, we remedied two shortfalls of Xue et al. (2019). Firstly, we included positional information (line number and the position of the word in the line) in the predictive modeling, to compensate for potential position effects (Pynte et al., 2008, 2009; Kuperman et al., 2010). We found that they were indeed important (*FI*s > 0.10) for predicting first fixation duration and gaze duration, but not for predicting regression and total reading time or fixation probability. Secondly, we enlarged our sample size by recruiting more readers. In spite of the changes, results were much the same: the neural nets approach was the most suitable one, and the key features again were *word length*, *word frequency*, *orthographic neighborhood density*, and *sonority score*.

Of course, there is still room for further improvement. Firstly, we used only two sonnets—so as not to strain readers—but for some predictors (e.g., *higher frequent neighbors*, *M* = 0.55, *SD* = 1.11) two short texts may not produce sufficient variation. In future studies, our findings should therefore be checked with more and different poems (Fechino et al., in revisions). Secondly, according to the multilevel hypothesis of the NCPM (e.g., Hsu et al., 2015; Jacobs et al., 2016b), many foreground and background features, especially on the interlexical and supralexical levels, also contribute to the highly complex literary reading process. Before we can include them in empirical eye tracking studies, we still have to identify, define, and classify them more reliably, though. However, existing classification schemes often overlap or are inconsistent or incomplete (cf. Leech, 1969). Certainly, there are some promising approaches to quantifying the occurrence of rhetorical figures (Jakobson and Lévi-Strauss, 1962; Jacobsen, 2006; Jacobs, 2015a, 2017, 2018a; Jacobs and Kinder, 2017, 2018; Gambino and Pulvirenti, 2018), but many questions remain open as regards, for instance, possible weightings. Thirdly, for predictive modeling we aggregated the eye tracking data over participants, which may inflate certain psycholinguistic effects (Kliegl et al., 1982; Lorch and Myers, 1990). However, in neural nets it is not possible to consider subject effects as a random effect like in linear mixed models (e.g., Baayen et al., 2008). To make model comparisons possible, we thus had to use the aggregated values for both approaches.

## Data Availability Statement

The datasets generated for this study are available from the corresponding author on request.

## Ethics Statement

The studies involving human participants were reviewed and approved by Department of Education and Psychology at the Freie Universität Berlin. The patients/participants provided their written informed consent to participate in this study.

## Author Contributions

SX carried out the experiment, analyzed the data, and wrote the first draft of the manuscript. JL modified the manuscript. AJ improved the manuscript. All authors have contributed to and approved the final manuscript.

## Conflict of Interest

The authors declare that the research was conducted in the absence of any commercial or financial relationships that could be construed as a potential conflict of interest.

## Footnotes

1 https://www.sr-research.com/experiment-builder

2 https://www.jmp.com/en_us/software/predictive-analytics-software.html

3 https://www.sr-research.com/data-viewer/

4 For the neural nets we used the following parameter set: one hidden layer with 3 nodes, hyperbolic tan (TanH) activation function; number of boosting models = 10, learning rate = 0.1; number of tours = 10. For standard least squares regression, we only specified the nine fixed effects (*lineNo.*, *wordNo.*, *wl*, *logf*, *on*, *hfn*, *odc*, *cvq*, and *sonscore*) and predicted each eye tracking parameter using the same nine predictors.

5 https://scikit-learn.org/stable/modules/feature_selection.html
